# Scaling-up of a novel, simplified MFC stack based on a self-stratifying urine column

**DOI:** 10.1186/s13068-016-0504-3

**Published:** 2016-05-10

**Authors:** Xavier Alexis Walter, Iwona Gajda, Samuel Forbes, Jonathan Winfield, John Greenman, Ioannis Ieropoulos

**Affiliations:** Bristol BioEnergy Centre (B-BiC), Bristol Robotics Laboratory, T-Block, Frenchay Campus, University of the West of England, Bristol, BS16 1QY UK; Microbiology Research Laboratory, Department of Biological, Biomedical and Analytical Sciences, Faculty of Applied Sciences, Frenchay Campus, University of the West of England, Bristol, BS16 1QY UK

**Keywords:** Partially submerged cathodes, Scaling-up, Ammonium abstraction, Microbial fuel cell stack, Bioenergy

## Abstract

**Background:**

The microbial fuel cell (MFC) is a technology in which microorganisms employ an electrode (anode) as a solid electron acceptor for anaerobic respiration. This results in direct transformation of chemical energy into electrical energy, which in essence, renders organic wastewater into fuel. Amongst the various types of organic waste, urine is particularly interesting since it is the source of 75 % of the nitrogen present in domestic wastewater despite only accounting for 1 % of the total volume. However, there is a persistent problem for efficient MFC scale-up, since the higher the surface area of electrode to volume ratio, the higher the volumetric power density. Hence, to reach usable power levels for practical applications, a plurality of MFC units could be connected together to produce higher voltage and current outputs; this can be done by combinations of series/parallel connections implemented both horizontally and vertically as a stack. This plurality implies that the units have a simple design for the whole system to be cost-effective. The goal of this work was to address the built configuration of these multiple MFCs into stacks used for treating human urine.

**Results:**

We report a novel, membraneless stack design using ceramic plates, with fully submerged anodes and partially submerged cathodes in the same urine solution. The cathodes covered the top of each ceramic plate whilst the anodes, were on the lower half of each plate, and this would constitute a module. The MFC elements within each module (anode, ceramic, and cathode) were connected in parallel, and the different modules connected in series. This allowed for the self-stratification of the collective environment (urine column) under the natural activity of the microbial consortia thriving in the system. Two different module sizes were investigated, where one module (or box) had a footprint of 900 mL and a larger module (or box) had a footprint of 5000 mL. This scaling-up increased power but did not negatively affect power density (≈12 W/m^3^), a factor that has proven to be an obstacle in previous studies.

**Conclusion:**

The scaling-up approach, with limited power-density losses, was achieved by maintaining a plurality of microenvironments within the module, and resulted in a simple and robust system fuelled by urine. This scaling-up approach, within the tested range, was successful in converting chemical energy in urine into electricity.

## Background

The Microbial Fuel Cell (MFC) is a technology in which microorganisms employ an electrode (the anode) as the end-terminal electron acceptor in their electroactive anaerobic respiration. This results in the direct transformation of chemical energy (reduced organic matter) into electrical energy, which in turn means that substrates such as wastewater or urine can be used as fuel. The first working MFC was reported by Potter in 1911 [1], and despite the long history of the technology, it is only in the last 20 years that significant breakthrough has been reported [2–5], which includes reactor design [6–10], component optimisation [11–14], and stack configuration [5, 15, 16]. Another major consideration inherent to the advancement of the technology, is the need to identify inexpensive materials, and there has been significant progress in this direction, making practical implementation a realistic proposition. In this respect, ceramics have become a cheap yet viable alternative material now used by a number of research groups that are developing MFCs [9, 17–19].

Amongst the various types of organic waste used as fuel for MFCs, urine is of interest since it can be employed undiluted as an energy source to power applications [20]. Moreover, the fact that urine represents 75 % of the nitrogen present in domestic wastewaters—yet accounts for only 1 % of the total volume [21]—supports the widespread idea of source-separated urine for a more efficient treatment of both urine and faecal matter [22, 23]. Most of the nitrogen in fresh urine is in the form of urea. However, urea gets quickly hydrolysed (within hours) by bacterial urease into ammonium and bicarbonate [24]. In this respect, MFCs have been shown to efficiently treat the ammonia present in urine, whilst producing electrical energy by the consumption of organic matter [25, 26]. All these studies were carried out in compartmentalised MFCs, and the ammonium abstraction was mainly driven by the pH difference between the anodic and cathodic compartments. In these reports, the ammonium migrates from the anode to the cathode through the cation exchange membrane, and production of hydroxyl at the cathode increases the pH, resulting in the formation of gaseous ammonia that subsequently escapes the system [25, 27]. In some specifically designed set-ups employing biocathodes, a denitrification process occurs, and the nitrogen escapes as di-nitrogen [28, 29]. Moreover, recent results have demonstrated that anaerobic electroactive ammonium oxidation could occur in the anodic compartment [30] and that such activity results in the accumulation of nitrate.

In order to scale-up the MFC technology towards out-of-the-lab applications, a number of approaches are being pursued. One method is the stacking of small-scale MFC units for useful electricity generation [5]. Hence, the implementation of MFC technology implies (1) a plurality of units, and (2) grouping of the units into series and parallel electrical connections. For this to work, each unit needs to have a simple design allowing easy replication and multiplication to build a stack. It has previously been shown with the use of a ceramic plate that an anode and a cathode can share the same liquid electrolyte [31]. Based on this, and to simplify a collective system, the MFC design presented here was developed with the aim of all sub-units within each module being connected in parallel, since they would be subject to the same embodiment and exposed to the same liquid electrolyte. This allows for the production of larger units that would then be electrically connected in series.

In MFCs, electroactive populations develop in biofilms and form exchange interfaces [32] that can also act as a bio-exchange membrane between the anodes and cathodes [33]. The principle behind the present design was to increase the area of these exchange interfaces within a defined volume. At the macro-scale (>250 mL), single units have diffusion limitations at the scale of the half-cell compartment and at the scale of the electrode itself. In the first case, the performance of the MFC is limited by the diffusion of anolyte/catholyte (reservoir) to the anode/cathode (consumption). For the same reason, thick anodic materials (of large MFCs) would limit the diffusion of nutrients towards the centre of the electrode, thereby limiting the levels of performance; this holds true especially for biofilm communities. In general, MFCs with high surface area of exchange to volume ratios are more power dense than MFCs with small surface area to volume ratios [5, 34, 35]. In other words, smaller MFCs are more power dense than larger ones. Hence, the approach in the present study was to enlarge the unit size whilst increasing bio-interfaces at a fixed ratio, in order to maintain a similar power density to that generated by smaller units. Such stack systems naturally comprise a collective of these interfaces, characterised by many microenvironments. Having multiple microenvironments means that increasing unit size does not increase diffusion limitations, since at the electroactive scale, all interfaces and diffusion distances would be kept to a minimum. In the present study, we demonstrate the results of membraneless MFC stacks, in which the anodes and cathodes are exposed to the same electrolyte (urine) as a self-stratifying column (SSC-MFC). This design relied on the self-stratification of a liquid under the natural activity of the microbial consortia thriving in the system. The MFC module size was increased from 900 to 5000 mL via compartmentalised multiplication, increasing total power output whilst maintaining similar power densities.

## Methods

Two different configurations were employed in this study; the first was a tubular reactor configuration that was used to investigate whether anodes and cathodes could operate in the same liquid medium and the second were boxed reactors designed to optimise surface area to volume ratio and power density.

### Tubular reactor configuration

**Fig. 1:**
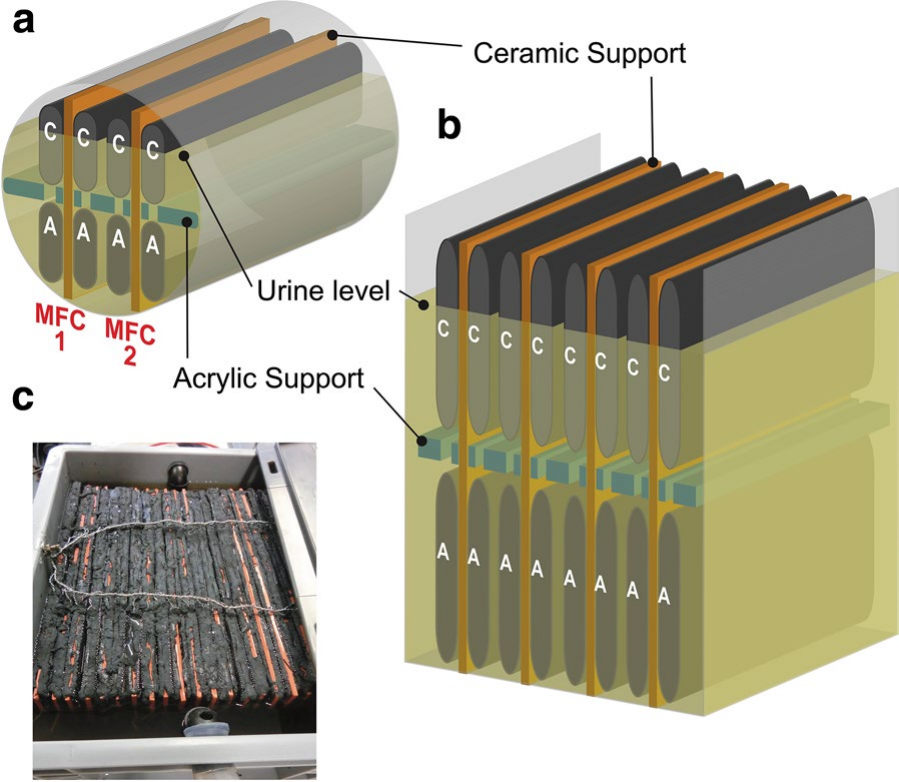
Illustration of the self-stratifying column membraneless microbial fuel cells (SSC-MFC). **a** schematic of a cross section of a tubular SSC-MFC stack comprising two elongated ceramic supports. **b** schematic of a rectangular SSC-MFCs with one out of two ceramic supports removed. In both **a, b**, the “*C*” and “*A*” stand for cathode and anode, respectively. **c** top view of the mounted stack comprising 20 MFCs and 21 ceramic supports inserted in a 5 L box (M5–21)

A preliminary test was performed using a tubular reactor configuration to investigate the effects of two MFCs and all their electrodes being submerged in the same liquid (Fig. 1). This experiment used an MFC that consisted of two anodes and two cathodes surrounding a single ceramic support. Two self-stratifying urine column MFCs (SSC-MFC) were fitted into a PVC tube of 48-mm diameter. Each SSC-MFC consisted of a 2-mm thick ceramic support (3.5 × 12 cm; Fig. 1a) surrounded by two cathodes on the upper-half and two anodes on the bottom-half; a perforated acrylic sheet (2 mm thick) was used to separate the anode from the cathode. The perforations were 2 mm wide, 120 mm long and spaced 5 mm apart. The main function of the acrylic separator was to prevent any physical contact between anode and cathode, thus avoiding any electrical short-circuit between them. Nonetheless, the perforations in the separator allowed the electrolyte to circulate. The cathodes were made from a mixture of activated charcoal powder (G.Baldwin & Co, London, UK) and polytetrafluoroethylene (60 wt% dispersed in H_2_O, Sigma-Aldrich, UK) spread over a 30 % PTFE treated carbon veil support (1.5 × 12 cm; PRF Composite Materials Poole, Dorset, UK) [36]. The displacement volume of urine was 60 mL and the liquid level reached 75 % of the cathode height (i.e. 75 % was submerged and the remaining 25 % was exposed to air). The anode consisted of 20 g m^−2^ carbon veil (PRF Composite Materials Poole, Dorset, UK): each unit comprised a 250 cm^2^ surface area folded into a 1.5 cm × 12 cm projected surface area. Each MFC had two of these 250 cm^2^ anodes, one on each side of the ceramic support (Fig. 1a). The two MFCs had independent electrical connections, but shared the same electrolyte. The applied load was initially fixed at 600 Ω, which was consistent with previous set-ups of similar electrode surface area.

### Boxed reactors configuration

#### Small rectangular stack (M0.25–6)

From the results of the tubular test-MFC, a small rectangular box was constructed in order to optimise the surface of exchange in a stack comprising flat MFCs (Fig. 1c). The performance of this stack was also used to compare power density with the larger stack described in the next section. This stack was built with the objective of having a scalable design towards ease of practical implementation: simple to build and configure (electrically and hydraulically) and of minimal maintenance (one inlet and one outlet per stack).

This small stack consisted of six SSC-MFCs (M0.25–6), all connected in parallel. These test units comprised six elongated supports—12-cm long, 9-cm height and 2-mm thickness—with an exchange volume of 250 mL (M0.25–6), in an attempt to optimise fuel percolation between anodic and cathodic depths, and to maintain homogeneous redox potential gradients at the biofilms’ level [37, 38]. Similar with the tubular reactor, the 6 anodes were below and six cathodes were above the support. Each anode was made from 600 cm^2^ carbon fibre veil electrode (20 g m^−2^; PRF Composite Materials Poole, Dorset, UK), folded into 10 × 4.5 cm projected surface area. The cathode consisted of a stainless steel mesh (10 × 3.5 cm), serving as the current collector, on which a layer of activated-carbon charcoal was spread (8 ± 0.1 g). This paste was of a mix of 5 ± 0.05 g activated carbon powder, 1.9 ± 0.1 mL of polytetrafluoroethylene solution (PTFE; 60 wt %, Sigma-Aldrich, UK) and 10 mL of water. Each cathode had a final size of 10 × 4 cm.

#### Large stacks: 5 L, 21 separators (M5–21) or 11 separators (M5-11)

In order to create a system more suitable for powering real-life applications, a larger unit was designed to accommodate the volume of urine produced daily by 1-3 individuals (2.4 ≤ Vol ≤ 6 Ld^−1^). This larger unit (M5–21) was the scale-up, in the x and y dimension, of the M0.25–6. The M5–21 stack consisted of 20 MFCs connected electrically in parallel and held by 21 vertical ceramic supports: each MFC had the same height and thickness as in M0.25–6 but twice the length (Fig. 1c).

To this end, 5-L rectangular plastic “euro stacking” containers (12 × 17 × 27 cm) were chosen as the housing for each MFC stack (Plastor Limited, Berkshire, UK). The first box was assembled with 21 separators making up 20 MFCs (as defined in the previous paragraph; the first and last supports did not have an anode on the side facing the box), each of 21-cm length (Fig. 1c). All anodes (*n* = 20) and cathodes (*n* = 20) of the MFCs contained in the same boxes were electrically connected in parallel. Each anode consisted of 1500 cm^2^ carbon fibre veil electrode (20 g m^−2^; PRF Composite Materials Poole, Dorset, UK), folded into 20 cm × 4.5 cm projected surface area. Each cathode consisted of a pair of stainless steel mesh (21 × 3.5 cm), serving as the current collector, on which a layer of activated carbon was spread (16 g ± 0.1 g) prepared in the same way as for the M0.25–6 box. Each cathode sub-unit had a final size of 21 × 3.8 cm and 2-mm thickness. All anodes and cathodes were configured in pairs around each ceramic support, and interconnected with stainless steel wire. Each ceramic support consisted of a pair of 9 × 10.5 cm terracotta sheets (2 mm thick). The displacement volume was of 1.5 L for the M5–21 box. Based on results, the units employed in the cascade were assembled with only 11 ceramic supports (M5–11; Fig. 1b) instead of 21 (M5–21). Although the displacement volume of the M5-11 was higher (1.75 L), the two types of box had the same surface areas of anode and cathode in total, i.e. 30,000 and 3612 cm^2^, respectively.

### MFC operation and monitoring

#### Feedstock and MFC maturation

The MFCs were inoculated with anolyte effluent removed from an established MFC that had been running under long-term continuous flow conditions of urine. Urine (pH between 6.4–7.4) was collected anonymously from healthy individuals with no known previous medical conditions, on a daily basis, and pooled together. Following the inoculation period, the subject MFCs were fed with urine from a collector tank. The urine age ranged between 1 and 3 days when fed to the MFCs. The MFCs were operated in ambient temperature conditions (22.0 ± 0.5 °C). All MFC designs were pulse-fed, either by the use of a pump (tubular reactor and M0.25–6 box), or by the use of a syphon (M5–21/M5–11), which allowed for mixing of the stratified urine column and periodic replenishment of fuel.

#### Data capture and polarisation experiments

Voltage output was monitored against time using an Agilent LXI 34972A data acquisition/switch unit (Farnell, UK). Measurements were recorded every minute. Recorded raw data were processed and analysed using Sigma Plot v11 software (Systat Software Inc, London, UK). The polarisation scans were performed, using an automated resistorstat [39], and the resistive load values ranged from 38,000 to 4 Ω. Each resistor was connected for a period of 10 min. The resistorstat was not accurate at resistances lower than 10 Ω, which corresponded to the optimum load for the M5–21 and M5–11 units. Therefore, for the M5–21 and M5–11 units, the polarisation experiments were complemented by manually sweeping 57 resistor values covering the range 30,000–1 Ω. The time interval between resistance changes was as before 10 min.

#### Ammonium measurements

The ammonium concentration profiles were performed in the geometrical centre of the M5–11 stack. Samples of 100 µL were taken at 5 depths (-2, -18, -34, -52 and -64 mm) and directly diluted 10×. These dilutions were then carried further to a final dilution of 10^4^. Ammonium concentrations were determined immediately after sampling using test kit reagents for ammonium (Lovibond, Amesbury, UK), and analysed using a Camlab MC500 colorimeter. A sample was taken from each of the five different depths and analysed. This was repeated three times so as to obtain triplicate samples from each depth. Ammonium concentration gradients were converted into flux units employing Fick’s law of diffusion using the following equation:

1 $$J_{{{\text{NH}}_{ 4} }} = D_{{{\text{NH}}_{ 4} }} \left( {\frac{\Delta C}{\Delta Z}} \right)$$

where *J*_NH4_ is the net flux of the ion µmol.cm^−2^ s^−1^; *D*_NH4_ is the diffusion coefficient of ammonium (2.09 × 10^−5^ cm^−2^ s^−1^) [40]; ∆*C* is the concentration gradient in µmol cm^−3^; and ∆*Ζ* the distance, in cm, between the two points. For ease of calculation, it was assumed that the media, through which ammonium was diffusing, was only liquid and the porosity and tortuosity of carbon veil were ignored. The total ion flux across the stack was estimated by multiplying the calculated flux by the surface area of the liquid phase of the stack using Eq. (2), defined as following:

2 $${\text{SA}}_{\text{liquid}} = {\text{SA}}_{\text{stack}} {\mathbf{ - }}\left( {{\text{SA}}_{\text{ceramic}} + {\text{SA}}_{\text{cathode}} } \right)$$

where SA_liquid_ is the surface area of the liquid phase (cm^2^); SA_stack_ is the total surface area of the stack (cm^2^); SA_ceramic_ is the cumulative surface area of all the ceramic support (cross section, cm^2^); and SA_cathode_ is the cumulative surface area of the cathodes (cross section, cm^2^).

## Results and discussion

### Independent electrical behaviour of MFCs submerged in the same electrolyte

**Fig. 2:**
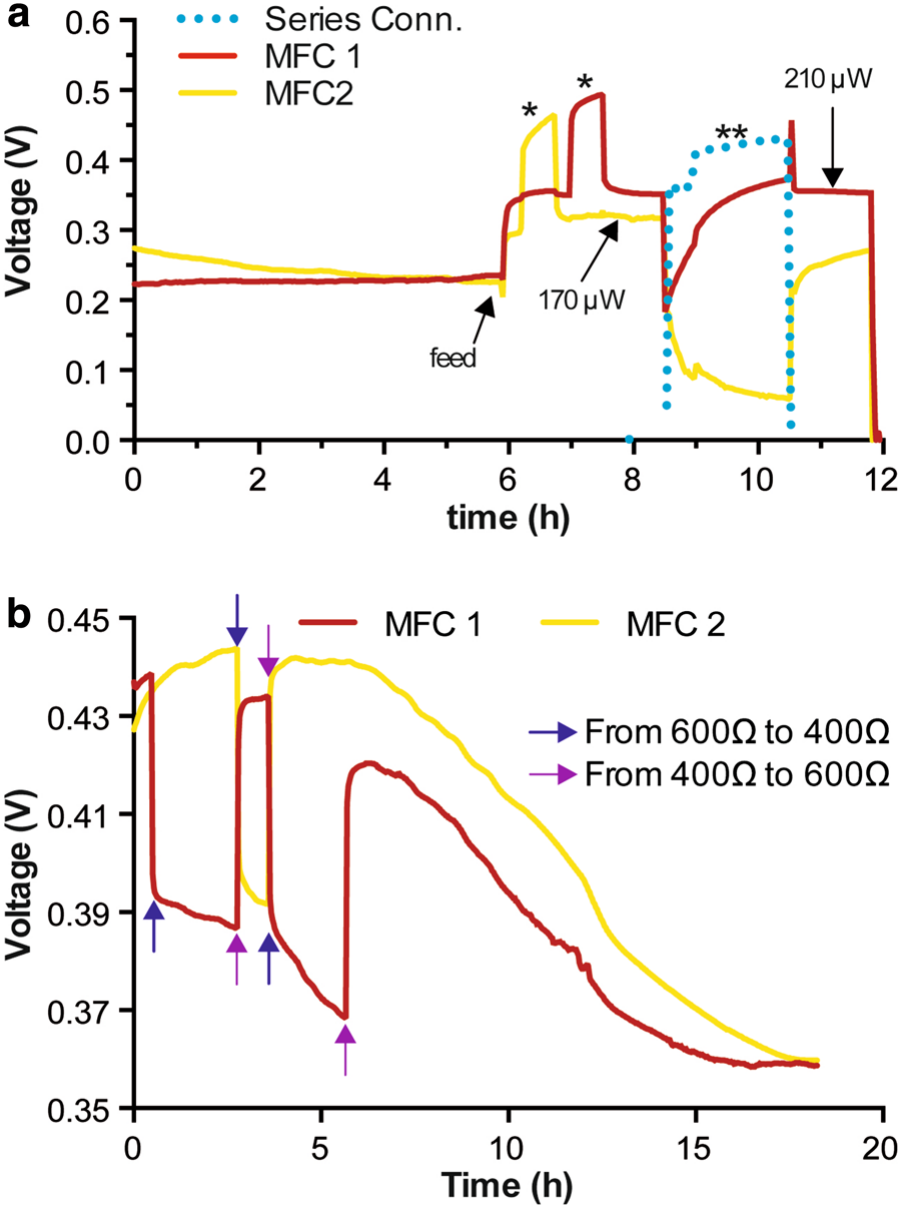
Voltage monitoring of two independent MFCs submerged in the same liquid. Each MFC was loaded with a 600-Ω resistor. **a** *Star* indicates when only one of the two MFCs was put in open circuit. The *blue dotted line* is the voltage monitoring when the two MFCs were electrically connected in series *(double star*, 1200 Ω resistor). **b** The *arrows* indicate when the resistive loads were swapped between 600 and 400 Ω. Data show the independent electrical behaviours of both MFCs

The two MFCs, of the tubular MFC, were initially connected electrically in parallel and results showed that up to 500 µW of power was generated (data not shown), despite the anodes and cathodes sharing the same electrolyte and only being 2 mm apart from each other. The question that arose from this was in relation to the ‘boundary’ that would constitute an individual microbial fuel cell. Thus, the electrical connection of the test-MFC was such that each MFC (Fig. 1a), was electrically independent. The voltage of each MFC was then monitored under separate, variable resistive loads. Results demonstrated that the two MFCs displayed independent behaviours when different loads were applied to them (Fig. 2). It is worth noting that the optimum urine fluid level i.e. ¾ submerged cathode was empirically found after testing different configurations: ¼ submerged cathode (¾ exposed to air) gave lower but stable performance and a completely submerged cathode initially gave a higher output, but collapsed after only 2 h (eventually decreasing to zero).

Connecting the two MFCs in series resulted in an instant drop in power from one of the MFCs (Fig. 2a), emphasising that in this set-up, the MFCs can only be electrically connected in parallel. Nonetheless, if the definition of an MFC, as stated above, is valid, then this notion can be pushed further. The space between the two MFCs was only 3–4 mm; hence, the distance between their respective neighbouring anodes and cathodes was short. The two MFCs showed nevertheless different electrical behaviours, although not entirely independent. This demonstrates that what was considered as a single MFC unit of two cathodes and two anodes surrounding a single ceramic support was in fact two MFCs electrically connected in parallel, and the series connection behaviour suggests that there is a temporal element connected with fully independent MFC units. Based on this, and considering the dynamic bridging behaviour at the molecular level, which is time- and fluid flow-dependent, it was determined that in this set-up, where a batch of MFCs shares the same electrolyte (i.e. preventing series connection), an individual MFC consisted of a single cathode above a single anode.

### Feeding rate of self-stratifying MFC systems

#### Small rectangular stack: 0.25 L, six separators (M0.25–6)

**Fig. 3:**
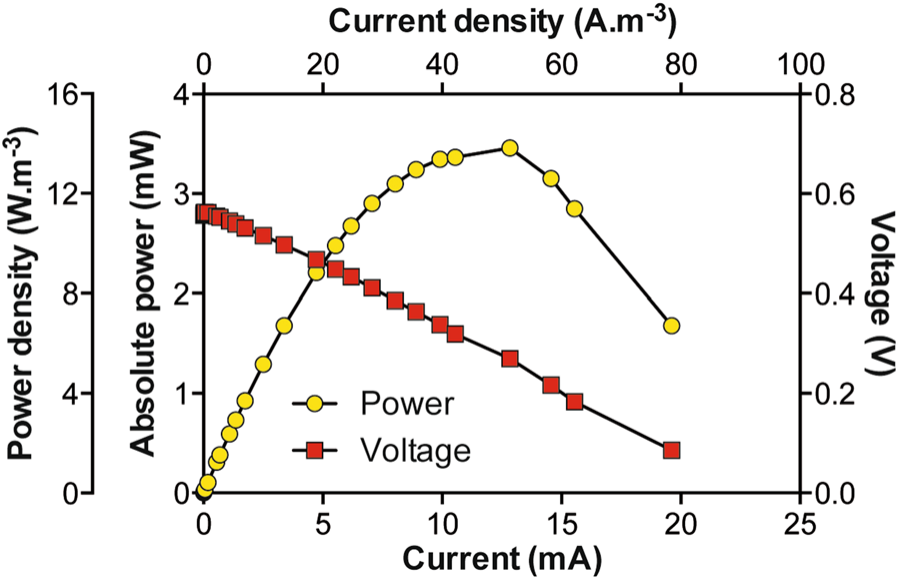
Polarisation and power curves of the stack comprising six MFCs connected in parallel. The resistive load values ranged from 38,000 to 4 Ω. Each resistor was connected for a period of 10 min. The volumetric power densities were obtained by normalising the power by the displacement volume of the whole reactor (250 mL)

Normalised to the total displacement volume of 250 mL, this stack of a simple design was characterised by a high power density of ≈14 W/m^3^ at ≈380 mV (Fig. 3). The implementation of a fuel-based biotechnology implies that the feeding rate is adapted so that steady-state conditions are maintained in terms of power. After 1 week, the M0.25–6 reached steady power production levels under semi-continuous feeding (replenishment of media once every day). The M0.25–6 was then subjected to various feeding rates by connecting an automated feeding system (pump + timer). As discussed below, the semi-continuous flow was not adapted to the design of the MFC.

**Fig. 4:**
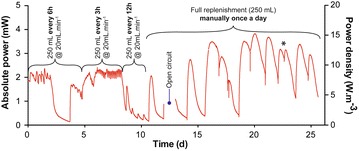
Power produced by a stack of six MFCs connected in parallel. Data illustrate the influence of the feeding regime on the stack behaviour. The resistive load was 40 Ω. *Asteris*k indicates that the stack was manually replenished twice during the same day. The power drop from day 2 to 3 was due to the drowning of the cathode (mechanical blockage)

In this line of experiments, four feeding regimes were implemented, which were empirically determined, and also were subject to practical considerations (i.e. continuous pump flow vs a single exchange of liquid volume). Both the 3 and 6-h pulse-feeding regimes were characterised by a stable absolute power output of ~2 mW (Fig. 4). The drop in power, observed between days 2 and 4 was in fact due to a mechanical failure in the form of blockage and concomitant “drowning” of the cathode (i.e. fully exposed to the liquid bulk instead of being partially exposed to O_2_). When the 12-h regime was introduced, power levels dropped, which suggested that the supply rate was not sufficient to support stable output. However, results from the 24-h regime contradicted this because steady-state power output conditions were observed, indicating that daily replenishment of the medium was sufficient (Fig. 4). This suggests that the limitation observed under the 12-h regime was due to some factor other than the pulse-feed rate. The flow rate of the pump could not exceed 20 mL min^−1^, and the replacement of the whole electrolyte occurred only after 12 min 30 s. Conversely, the 24-h regime was done manually with the removal of all the electrolyte prior to replenishment. Therefore, the results tend to confirm that a flow rate of 20 mL min^−1^, even if 250 mL was pumped in, was insufficient to obtain a homogeneous replacement of the electrolyte, resulting in a power-output limitation. Because of this heterogeneous mixing, some-to-most of the “fresh” added fuel was leaving the system without being consumed, and as spent fuel accumulated towards the bottom of the column, such as in natural stratified bodies (i.e. meromictic water column, lakes). Under the 24-h pulse-feed regime, the system was able to reach higher absolute power of ~3.8 mW (Fig. 4). These results emphasise that this MFC system requires a pulse-feed regime with a high flow rate (>20 mL min^−1^) in order to ensure homogeneous replenishment of the electrolyte. However, understanding better the feedstock replenishment rate, as suggested by the need of thorough mixing, requires further investigation.

#### Larger stack: M5–21 and M5–11

 Based on the above and in order to have a homogeneous mix of the electrolyte, the syphon principle was employed, which provided a fixed quantity in bursts of high flow rate (≈4 L min^−1^). The syphon mechanism would also allow the whole system to better adapt to variations in inflow rates thus ensuring a consistent supply of fuel (urine).

**Fig. 5:**
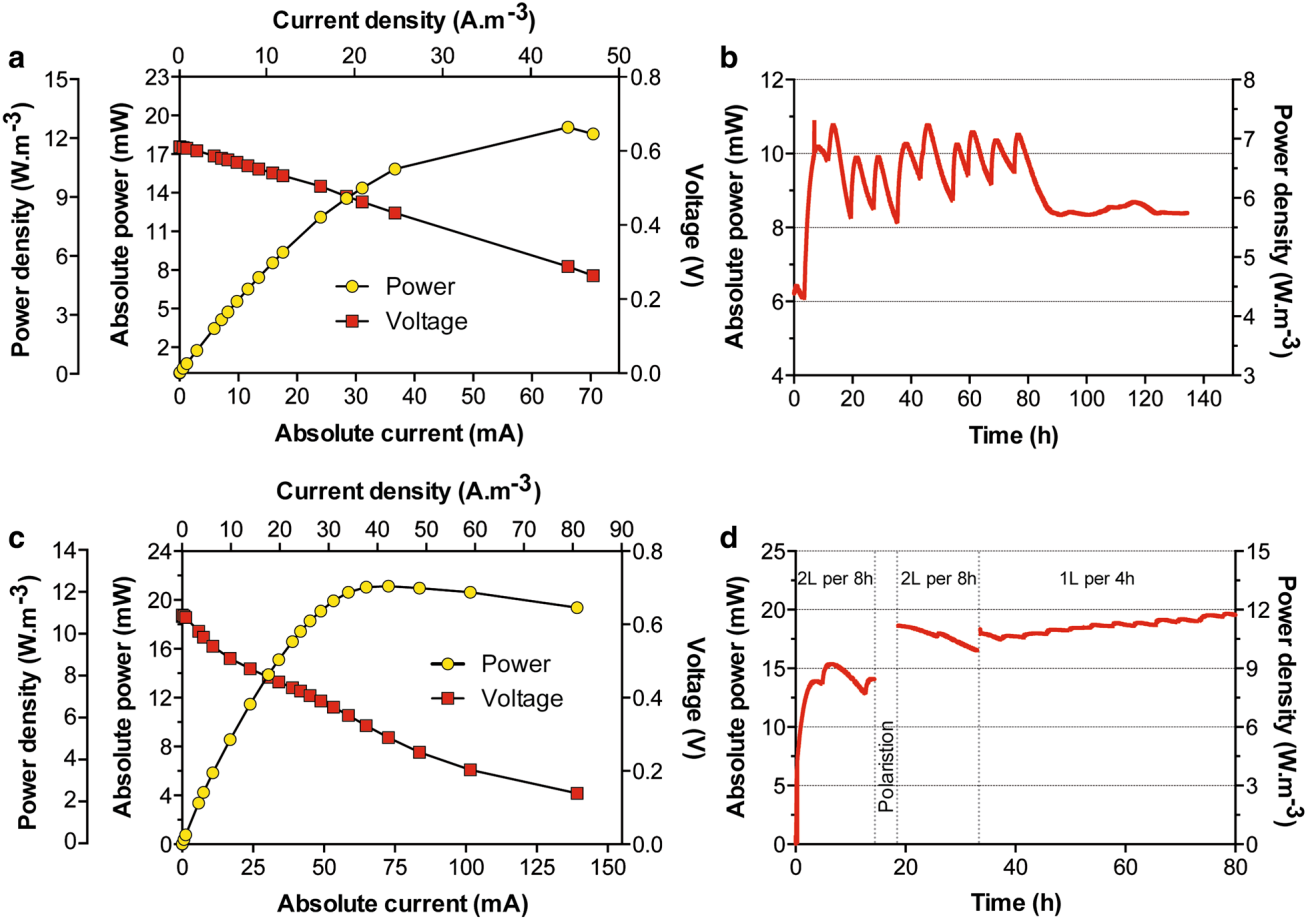
Electrical characterisation of the M5-21 SSC-MFC unit (**a, b**) and the M5–11 SSC-MFC unit (**c, d**). **a** Polarisation and power curves. The resistive load values ranged from 38,000 to 4 Ω. Each resistor was connected for a period of 10 min. Power normalised by total displacement volume (1.5 L); **b** Temporal power production under a 2 L 8 h^−1^ feeding regime. **c** Polarisation and power curves. The resistive load values ranged from 30,000 to 1 Ω. Each resistor was connected for a period of 10 min. Power normalised by displacement volume (1.7 L). **d** Temporal power production under two feeding regimes, with an identical daily rate

A polarisation sweep was performed on the stack (Fig. 5a), and the maximum power point was higher (≈20 mW) than the output produced (≈11 mW) during stable running conditions (Fig. 5b) under the equivalent load (9 Ω). In preparation for the polarisation run, the system was allowed to settle under open-circuit conditions as fresh urine was fed into the stack. The polarisation sweep only lasted 4 h, which was half the hydraulic retention time (HRT) of the stack under running conditions. Therefore, it is safe to assume that the nutrients were not depleted by the end of the polarisation experiment. In addition, results from the M0.25–6 stack indicate that the 8-h HRT, under which the M5–21 stack was operated, should have been sufficient for stable current production. Therefore, it was hypothesised that the discrepancy between the power produced under polarisation compared to that under running conditions was due to an insufficient mixing of the fuel.

To test the hypothesis that the stack was too densely packed for allowing the fresh fuel to be homogeneously mixed, half of the ceramic supports were removed, leaving a 2-mm channel between one of the two anodes and cathodes. This stack was designated as the M5–11 SSC-MFC unit because it now had 11 ceramic dividers as opposed to 21 used previously. Using this new configuration, a polarisation sweep produced the same maximum power as the previous, more packed, design (Fig. 5c).

**Fig. 6:**
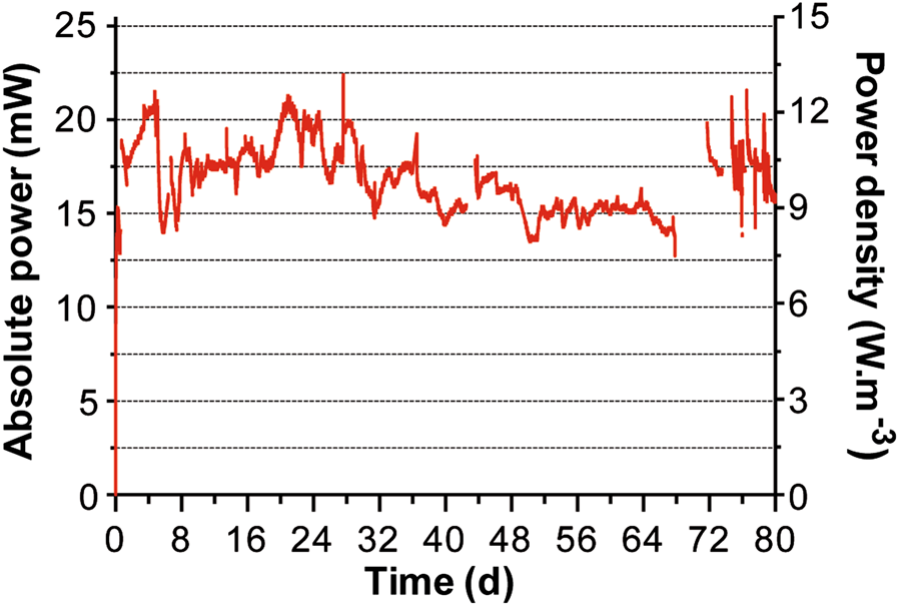
Power produced by the SSC-MFC stack of 20 MFCs connected in parallel (M5–11). Data illustrate the stability of the stack behaviour over the 80 days of the experiment. The resistive load was 9 Ω during the first 44 days. After that, the unit was connected in series with other MFC stacks under various loads and conditions, and hence, the noticeable variability of the produced power

During the first 18 h, the M5–11 produced more power, than the M5–21 design, and reached an absolute power of 15 mW (Fig. 5d). This verifies that the power limitation under running conditions, for the M5–21 design was due to a heterogeneous mixing during fuel replenishment and not nutrient depletion. The power decreased over the next 20 h illustrating that the feeding rate (2 L 8 h^−1^) was not optimal (Fig. 5d). Following the hypothesis that the nutrients for electroactive anaerobic respiration were not limiting, but the electrolyte mixing was, a new feeding rate of 1 L 4 h^−1^ was applied, which maintained the same daily feeding rate (6 L d^−1^). At this feeding rate, the stack was able to reach a maximum power output of 21.5 mW (Fig. 6), during the first 3–4 days. After 8 days, the stack produced approximately 17.5 ± 2 mW. After the 40th day, the stack was connected to other units in cascade, in order to investigate the behaviour of this design under a series electrical connection (Fig. 6).

### Ammonium vertical stratification

**Fig. 7:**
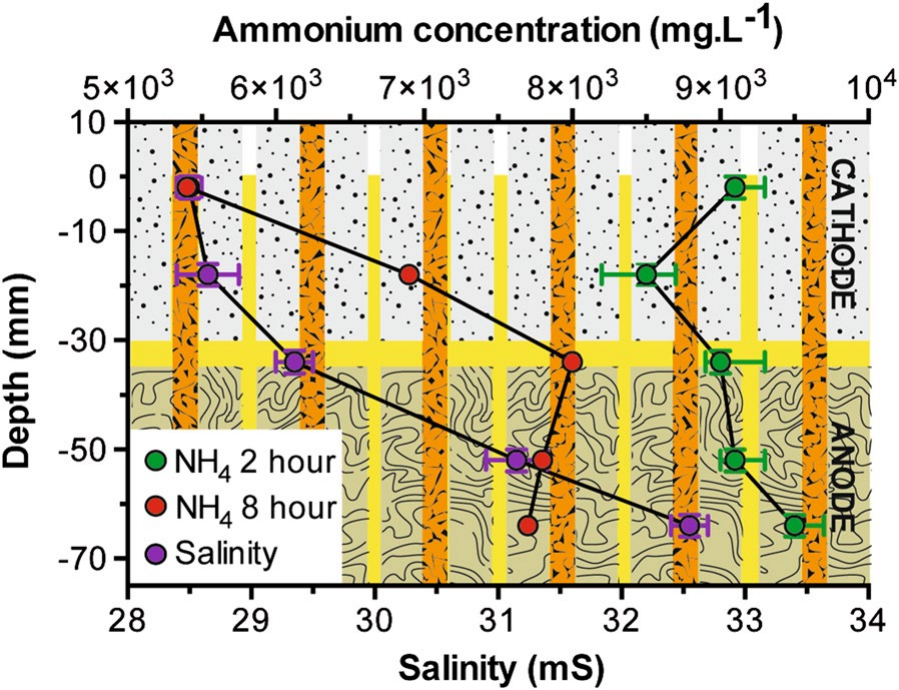
Vertical profiles, within the M5–11 stack, of electroconductivity and of ammonium concentrations. The 0 mm depth indicates urine level. *Green* and *Red circles* are two different stratification periods. *Green circles* show the median of triplicates and *error bars* indicate the lowest and highest measured values. *Red* *circles* are single data points. The hours shown, indicate the time spent between the last feed and the ammonium analysis

The anodes and cathodes shared the same electrolyte with only 5 mm of urine separating each other (Fig. 1c). In the MFC described here, it is the chemical stratification that allows both electrodes to function as cathodes and anodes. The electroconductivity profiling of the “water-column” (or urine-column), performed at the same time as the analysis, indicated two gradients that corresponded to both the anode and cathode. These measurements confirmed that the electrolyte was vertically stratified (Fig. 7), as typically occurs in natural water columns (e.g. lakes). However, the stratification was weak since neither a thermal nor a pH gradient was observed (data not shown). Early measurements of oxygen concentration showed that at a depth of 2–4 mm, the electrolyte was completely anoxic, suggesting that oxygen was not the dominant electron acceptor in the liquid phase. Moreover, since a fully submerged cathode was not sustainable, having part of the electrode open to air was vital for operation (optimally ¼ of the cathode area exposed). The hypothesis emerging from these observations was that part of the cathode operation is ultimately in the nitrogen redox cycle. To support this, ammonium and nitrate concentration profiles were determined (Fig. 7).

No nitrates were detected, and the ammonium concentration profiles showed a clear distinction between the cathodic and anodic parts (Fig. 7). It is worth noting that the third point of measurement was just in-between the anodic and cathodic parts of the electrolyte. On the one hand, the ammonium concentrations were relatively homogeneous in all the anodic layers of the electrolyte (bottom last three points = 9255 ± 271 mg L^−1^ NH_4_; green circles, Fig. 7). On the other hand, a depletion of ammonium was occurring in the middle of the cathodic part (8466 ± 251 mg L^−1^ NH_4_; green circles, Fig. 7). When the SSC-MFC was left unfed for a period of time exceeding the replenishment period, the ammonium concentration profiles indicate that the gradient extended from the bottom part of the cathode to its upper part (red circles, Fig. 7), 444 and 300 mmol L^−1^ NH_4_, respectively (8000 and 5400 mg L^−1^). This spatio-temporal behaviour suggests that a process of ammonium abstraction was initiated within the cathodic part of the “urine column” and not at its oxycline, i.e. at the urine/air interface. However, the 2- and 8-h concentration profiles were not measured from the same feed cycle. Therefore, to confirm that the 8-h ammonium profile was the evolution of the 2-h one, the gradients of ammonium concentration were converted into fluxes and compared.

The 2-h concentration profile displayed two gradients (green circles, Fig. 7): one from the surface towards the middle of the cathode and a second one from the bottom of the cathode towards the middle of the cathode, 38.89 and 33.33 µmol cm^−3^, respectively. The conversion of these gradients using Fick’s law of diffusion (Eq. 1) resulted in two ammonium fluxes of 5.08 × 10^−4^ µmol cm^−2^ s^−1^ and 4.35 × 10^−4^ µmol cm^−2^ s^−1^, respectively. The 8-h gradient (144.44 µmol cm^−3^), extending from the cathode lower part to its higher part, corresponded to a flux of 9.43 × 10^−4^ µmol cm^−2^ s^−1^. The total daily ion flux across one box (×mmol box^−1^ d^−1^) was estimated by multiplying the calculated flux by the surface area of the liquid phase alone (Eq. 2; 245 cm^2^). The obtained upper and lower fluxes for the 2-h profile were 10.76 and 9.11 mmol d^−1^ per box, respectively. The combined fluxes gave a daily NH_4_ depletion of 19.87 mmol d^−1^ per cascading box. The calculated 8-h daily NH_4_ flux was of 19.97 mmol d^−1^ per box. As both 2- and 8-h profiles have similar fluxes, the hypothesis that the 8-h profile is the temporal evolution of the 2-h one is supported, which also implies a stable and continuous NH_4_ abstraction rate.

Results demonstrate that there is a NH_4_ abstraction process occurring in the cathodic part of the electrolyte. Moreover, results tend to support the hypothesis that the ammonium abstraction is initiated in the middle of the cathodic layer. Over time, the gradient extends from the bottom of the cathodic layer to the surface of the urine column. As no pH gradient was measured (from top to bottom: 8.85 < pH < 9.04), the stripping of nitrogen as NH_3_ was difficult to assess [27]. The pH (9.04) of the electrolyte was close to that of the ammonium pKa (9.25), and therefore ammonia stripping could have occurred. However, because conclusive evidence was not measured, the ammonia stripping is assumed to be the cause of NH_4_ abstraction. In any case, as the oxycline was about 2 mm from the surface, an aerobic nitrification could not have dominated the anaerobic part where the NH_4_ abstraction was initiated. Moreover, as no nitrates were detected, and as it was a cathodic ammonium abstraction, ammonium was not the electron donor of an electroactive respiration [30]. Hence, the bio-electrochemical process leading to this NH_4_ abstraction was not identified in the urine column. Moreover, the cathodes are still open to air (¼) and the presence of oxygen is integral to its operation. This then raises the question of the importance of an alternative electron acceptor and its contribution to power output. Because the functioning of the SSC-MFC design relies on two principles—the vertical stratification of the redox state of various elements, and the multiplicity of microenvironments maximising local horizontal gradients—any further work aimed at elucidating the process of NH_4_ abstraction, and/or identifying the cathodic electron acceptor, should be carried out at the biofilm level.

### Power production and series connection

The previous sections demonstrated that individual MFC boxes are capable of practical, stable power output and that complex sets of reaction are taking place during the production of bioelectricity. Another important consideration is how best to configure stacks in order to optimise volume and ultimately power density. Previous studies have shown that when individual reactors are enlarged, there is a loss in power density [5]. This is verified in the current study where the power density of the small stack (M0.25) was ~14 W/m^3^ which was higher than the ~12 W/m^3^ of the larger stack (M5–11; Figs. 3, 5c; Table 1). However, the power-density decrease due to the scaling-up of the MFCs (from 250 to 1700 mL) was not as pronounced as in other studies [5, 34]. For example, while the size was increased by a factor of 6, the drop in power density was only 9 % (Table 1) which differs from other studies where, for example, a 4.7-size increase resulted in a 14 % power-density drop (Table 1 [5]).

**Table 1:** Relationship between the size increase of MFC and the subsequent power-density decrease

	SSC-MFCs		Air cathode MFCs [5]	
	M0.25	M5	Small	Medium
Volume (mL)	250	1500	6.3	29.6
Power density ( W/m^3^)	13.836	12.596	0.440	0.378
Factor of volume increase	×6		×4.7	
Power-density decrease (%)	8.9		14.1	

**Fig. 8:**
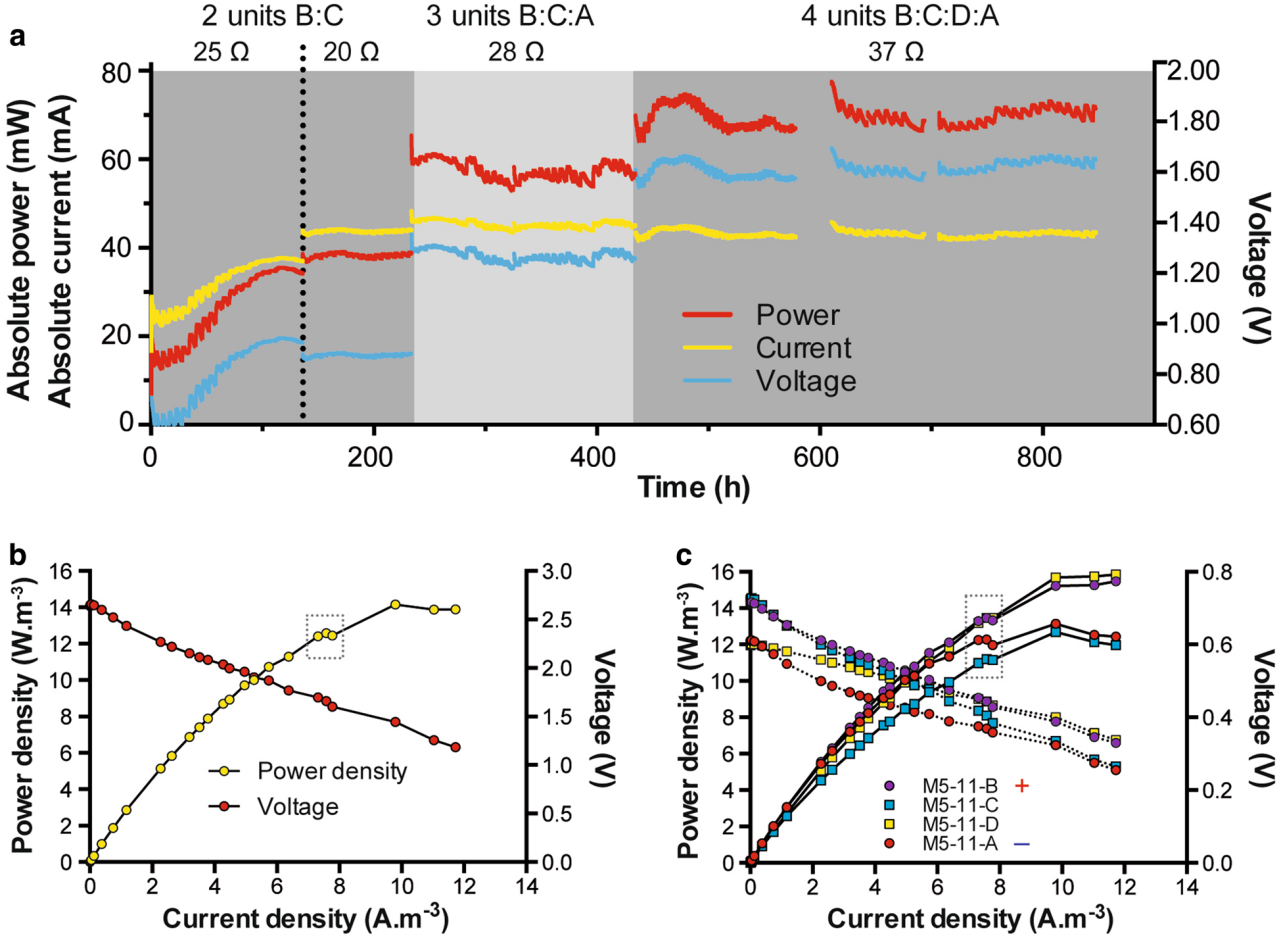
**a** Electrical outputs of the serial connections of increasing numbers of units in a single cascade. Resistances were set from the result of the first polarisation sweep. **b** Polarisation sweep of the cascade comprising four units connected in series. **c** Polarisation sweep of individual stack within the cascade. The *dotted squares* indicate the last points, prior to a non-planned refuelling, that are considered as the maximum power points. Each resistor, ranging from 30 k to 11 Ω, was connected for a period of 10 min

The scale-up in the current study focused on increasing the overall footprint, while at the same time maintaining microenvironments, so that the distances between electrodes were maintained, regardless of overall footprint. This resulted in less-pronounced mass-transfer losses compared to systems where each component has simply been enlarged in relation to the total volume. In addition, the close proximity of the electrodes submerged in the same fluid ensured there were fewer ‘dead spaces’ compared to previous reports of scale-up. Moreover, compared to previous set-ups with a relatively close design (benthic MFC), the power density of M5-11 stack was higher with 21.1 mW produced (Fig. 8b) for a total footprint volume of 5 dm^3^ instead of 36 mW for 30 dm^3^ [8]. When normalising to the total footprint surface (0.0459 m^2^), the power density of the M5–11 stack was 460 mW/m^2^, which is in the high range of existing systems employing more complex architectures [41] and even 1 order of magnitude higher than the most cost-effective single chamber system with similar volume (1.1 L; [28]). This is nevertheless not an accurate comparison, since these different set-ups employed fuels other than urine. Comparison with other MFCs employing urine as fuel indicates that the design developed here generated higher power densities: ~12.6 instead of ~10 [34], or 2.6 W/m^3^ [42]. This was all performed using inexpensive sustainable materials such as ceramics and a natural waste product—urine-as fuel.

Practical implementation of the MFC technology implies (1) plurality of units, and (2) configuration of units into series and parallel electrical connections. For these two reasons, the SSC-MFC design was developed with the aim that all sub-units, i.e. each box, would be connected in parallel with all the constituent MFCs sharing the same embodiment and electrolyte. This leads to the production of larger units that can be connected together electrically in series. Therefore, identical units to the M5–11 (M5–11A) were progressively connected in series, as they were built (M5–11B,C,D). All these boxes/stacks were assembled in a cascade manner, recycling the same feedstock at a pulse-feed rate of 1 L 4 h^−1^. There was no direct fluid bridging connection between each of the distinct boxes; in other words, there were air-gaps introduced between the outlet of the upstream box and the inlet of the downstream box.

Once the first cascade (M5–11B and M5–11C) reached steady state (Fig. 8a; 100 h) the increase in unit numbers resulted in a corresponding voltage increase: the current output was stable over time (~45 mA; Fig. 8a). This demonstrates that stacking individual MFC units can be achieved with stable results and without cell reversal. Polarisation experiments were carried out firstly on the four units connected in series (Fig. 8b) where a maximum power density of 14 W/m^3^ was achieved. To investigate the contribution of each box to the overall stack output, polarisation sweeps were performed on each of the four units at the same time. Figure 8c shows that the four units were comparable irrespective of their position in the cascade. Furthermore, the combined actual power from the four units was equivalent to that produced when they were connected together in series as a stack, and this shows the reproducibility and robustness of the system. Interestingly, during the polarisation sweeps, pumping of fresh urine occurred at the point in the sweep when the external resistances were reaching the lower values (21 Ω). This occurred after the points in the dotted boxes as shown in Fig. 8. The influx of fresh urine at these points, when the power should be dropping, resulted in an unexpected increase and hence in an extension of the power curve, and in fact, it demonstrated how healthy the biofilm was in this system. Further work will investigate the capabilities of the microbes at these lower resistances when they are fed with a steady stream of fresh urine.

## Conclusions

This novel MFC stack has all of its anodes and most of its cathodes submerged in the same electrolyte without short-circuiting. The rationale behind this design is that a phenomenon of horizontal self-stratification of a liquid feedstock column, i.e. urine, by microbial activity was occurring, similarly to what happens in natural environments colonised by microorganisms. Results have demonstrated that this phenomenon could be used to develop a novel and simplified type of membraneless MFC. Moreover, the stability and power density of this type of design are in the high range of existing more complicated designs. Due to the plurality of microenvironments in its macro structure, the SSC-MFC allows for the scale-up with limited losses in terms of power densities. This results in simplified stacks where all parallel units—consisting of a single cathode over a single anode—share the same embodiment. Results have shown that the performances were dependent of the pulse-feeding mechanism. Interestingly, an ammonium abstraction was measured in the anoxic part of the submerged cathodic layer. This anaerobic ammonium abstraction gives rise to the hypothesis that in such a set-up, part of the cathode operation is ultimately in the nitrogen redox cycle. Further investigations are needed to identify the process of electron and NH_4_ abstraction at the cathode.

## Abbreviations

M0.25–6: 250 mL displacement volume, 6 × ceramic supports
M5-21: 5000 mL displacement volume, 21 × ceramic supports
M5-11: 5000 mL displacement volume, 11 × ceramic supports
MFC: Microbial fuel cell
SSC-MFC: Self-stratifying urine column MFC
